# Transient Oxygen-Glucose Deprivation Causes Region- and Cell Type-Dependent Functional Deficits in the Mouse Hippocampus *In Vitro*

**DOI:** 10.1523/ENEURO.0221-21.2021

**Published:** 2021-09-28

**Authors:** Paul Grube, Cedric Heuermann, Andrei Rozov, Martin Both, Andreas Draguhn, Dimitri Hefter

**Affiliations:** 1 Institute for Physiology and Pathophysiology, University of Heidelberg, Heidelberg 69120, Germany; 2 Federal Center of Brain Research and Neurotechnologies, Moscow 117997, Russia; 3 RG Animal Models in Psychiatry, Central Institute of Mental Health, Mannheim 68159, Germany

**Keywords:** brain slices, hippocampus, hypoxia-ischemia, oxygen-glucose deprivation, parvalbumin interneurons, sharp wave-ripple oscillations

## Abstract

Neurons are highly vulnerable to conditions of hypoxia-ischemia (HI) such as stroke or transient ischemic attacks. Recovery of cognitive and behavioral functions requires re-emergence of coordinated network activity, which, in turn, relies on the well-orchestrated interaction of pyramidal cells (PYRs) and interneurons. We therefore modelled HI in the mouse hippocampus, a particularly vulnerable region showing marked loss of PYR and fast-spiking interneurons (FSIs) after hypoxic-ischemic insults. Transient oxygen-glucose deprivation (OGD) in *ex vivo* hippocampal slices led to a rapid loss of neuronal activity and spontaneous network oscillations (sharp wave-ripple complexes; SPW-Rs), and to the occurrence of a spreading depolarization. Following reperfusion, both SPW-R and neuronal spiking resumed, but FSI activity remained strongly reduced compared with PYR. Whole-cell recordings in CA1 PYR revealed, however, a similar reduction of both EPSCs and IPSCs, leaving inhibition-excitation (I/E) balance unaltered. At the network level, SPW-R incidence was strongly reduced and the remaining network events showed region-specific changes including reduced ripple energy in CA3 and increased ripple frequency in CA1. Together, our data show that transient hippocampal energy depletion results in severe functional alterations at the cellular and network level. While I/E balance is maintained, synaptic activity, interneuron spiking and coordinated network patterns remain reduced. Such alterations may be network-level correlates of cognitive and functional deficits after cerebral HI.

## Significance Statement

Neurons can recover following transient hypoxia-ischemia (HI), which is crucial for the survival of brain tissue in the ischemic penumbra. Brain tissue recovered from stroke is characterized by aberrant network activity. Essential to fast network oscillations are fast-spiking interneurons (FSIs), which, due to their high energy demand, might be especially vulnerable to metabolic stress. Here, our data from murine hippocampal slices demonstrate impaired functional recovery of FSI after transient HI compared to pyramidal cells (PYRs). Concurrently, ripple oscillations re-emerge with reduced incidence and altered waveforms. Thus, the present study confirms increased functional vulnerability of hippocampal FSI to HI and reveals specific impairments of network activity.

## Introduction

Hypoxic-ischemic conditions of the brain such as stroke are one of the leading causes of death and morbidity (GBD 2019 Diseases and Injuries Collaborators, 2020). While severe hypoxia-ischemia (HI), as in the ischemic core, leads to rapid necrotic cell death, mild metabolic distress, as present in the penumbra, causes functional deficits with or without subsequent delayed apoptotic cell death (Mattson et al., 2000; Broughton et al., 2009; Şekerdağ et al., 2018). The acute functional changes in hypoxic networks may determine long-term functional outcome when this tissue survives (Guggisberg et al., 2019). Still, little is known about short-term effects of such reversible HI events on single cell and network activity. Better understanding of these processes might help develop therapeutic or preventive interventions in transient ischemia and stroke.

A phenomenon commonly observed in the ischemic penumbra is the occurrence of one or more hypoxia-induced spreading depolarizations (HSDs; for a detailed review please refer to Somjen, 2001; Dreier, 2011). This event results from the complete breakdown of the membrane potential because of insufficient ATP-driven pump activity and entails a dramatic shift of ions, including a massive influx of calcium. Interestingly, neurons recover their membrane potential and ability to fire action potentials (APs) on timely re-oxygenation (Müller and Somjen, 2000; Hefter et al., 2016; Revah et al., 2019). It is suspected that HSD represents an additional insult, and it can be seen as a hallmark of HI-induced neuronal damage (Dreier, 2011).

Stroke patients oftentimes suffer from cognitive decline and memory deficits (Pendlebury and Rothwell, 2009). As the hippocampus is a pivotal region for learning and memory formation, and its CA1 area is highly vulnerable to HI (Schmidt-Kastner and Freund, 1991; Schmidt-Kastner, 2015), we conducted our study in this brain area. Hippocampal network architecture is largely preserved in acutely prepared brain slices from rodents, enabling studying its function in a reduced preparation without confounding systemic effects (Aitken and Schiff, 1986; Schurr and Rigor, 1989; Croning and Haddad, 1998; Hefter et al., 2016). One important pattern of hippocampal network activity are sharp wave-ripple complexes (SPW-Rs), comprising propagating waves of increased activity with superimposed high-frequency network oscillations (∼200 Hz). These patterns are regarded as crucial for a range of behavioral and cognitive functions such as memory formation. SPW-Rs entrain neuronal APs with high temporal precision (Ylinen et al., 1995; Both et al., 2008), which may be an important prerequisite for transient, stable activation of place-encoding ensembles (Buzsáki and Draguhn, 2004; Buzsáki, 2015; Hainmueller and Bartos, 2018). SPW-Rs arise within the hippocampus from an interplay between excitatory pyramidal cells (PYRs) and inhibitory interneurons (Csicsvari et al., 1999; Nimmrich et al., 2005; Schönberger et al., 2014; Buzsáki, 2015). Of particular importance for such fast synchronization processes are fast-spiking parvalbumin+ interneurons (PV-IN; Ylinen et al., 1995). Importantly, these cells might be especially vulnerable to HI-induced functional loss and cell damage (Kann et al., 2014; Kann, 2016; Povysheva et al., 2019; Elzoheiry et al., 2020). At the network level, this may lead to decreased inhibition-excitation (I/E) ratio, disturbed activity patterns and altered coupling between cellular spikes and local field potential (LFP) oscillations.

In this study, we compared the activity of fast-spiking interneurons (FSIs) and PYRs during fast network oscillations (SPW-R). We employed a model of transient oxygen-glucose deprivation (OGD) and reperfusion in acute murine brain slices to simulate transient, mild HI. Our data from field potential, tetrode, and patch clamp recordings confirm the hypothesis that transient OGD leads to strongly reduced FSI activity in comparison to PYR. This coincided with a partial recovery of SPW-R.

## Materials and Methods

### Animals and tissue handling

All experiments were performed in accordance with Baden-Wurttemberg state law (licenses T100 and T01/19) and institutional guidelines. Wild-type (WT) C57BL/6 and mice, expressing tdTomato in PV-INs [B6-129P2-Pvalbtm1(cre)Arbr/Uhg:B6-Gt(ROSA)-26Sortm14(CAG-tdTomato)Hze; below abbreviated as PV-tdTomato], obtained from Charles River Laboratories of both sexes were killed on postnatal day (P)60–P90. For this, the animals were deeply anesthetized using CO_2_ and decapitated following loss of righting reflex. The head was immediately transferred into ice-cold artificial CSF (ACSF), and the brain was swiftly removed from the skull, trimmed, glued onto a cutting platform, and surrounded by cooled ACSF [2–6°C; saturated with 95% O_2_/5% CO_2_ (carbogen)]. Composition of ACSF was as follows: 124 mm NaCl, 3.0 mm KCl, 1.8 mm MgSO_4_, 1.6 mm CaCl_2_, 10 mm glucose, 1.25 mm NaH_2_PO_4_, and 26 mm NaHCO_3_, pH 7.4. Horizontal slices were cut at 450 μm thickness using a Leica V1200s vibratome (Leica Microsystems). Three slices containing the ventral to mid portion of the hippocampus were then halved, so that each hemi-slice contained a complete horizontal section of the hippocampus, and transferred to a Haas-type interface chamber and left to recover for at least 2 h at 32–33°C while they were perfused with carbogen-saturated ACSF (flow rate 1.5–2 ml/min).

### OGD

Baseline electrophysiological data were recorded as described below while slices were superfused with ACSF. Only slices spontaneously expressing SPW-R with amplitude > 0.2 mV and incidence >1 Hz were chosen for experiments. Of six hippocampal slices, a median of 2 [1.5; 2.5] slices was used from each animal. To model HI the perfusion was switched to an equimolar ACSF solution containing mannitol instead of glucose, saturated with an oxygen-free gas mixture of 95% N_2_/5% CO_2_. As soon as an HSD was observed in the LFP recording, perfusion was switched back to carbogen-ACSF.

### Electrophysiological recordings

#### Tetrode recordings

LFP and single unit discharges were recorded with one to four tetrodes placed in the CA1 and CA3 PYR layers. The tetrodes were made of four twisted 12.5-μm-diameter tungsten wires (California Fine Wire) and connected to a DPA-2FX amplifier (NPI Electronics). Signals were amplified 100×, low-pass filtered at 10 kHz, high-pass filtered at 0.3 Hz, and digitized at 20 kHz for off-line analysis (1401 interface and Spike-2 data acquisition program; CED). In five slices, additional ACSF-filled glass microelectrodes (tip diameter, 2–4 μm) were used for DC recording. These signals were amplified 100× (EXT10–2FX amplifier; NPI Electronics) and digitized at 20 kHz. Data were continuously collected from baseline recording (20 min), during OGD (5–10 min) until 60 min after SD.

LFP recordings were analyzed using custom-written MATLAB routines, adapted from Hefter et al. (2016). Briefly, SPW-Rs were detected as local maxima in 10-x downsampled, <50-Hz lowpassed signal. Ripples were detected as local maxima in 10-x downsampled, 100- to 300-Hz bandpass-filtered signal. Ripple properties were analyzed using Morlet wavelet transformation of time windows starting 33 ms before and ending 67 ms after the peak of each SPW. Maximum power between 140 and 320 Hz was detected; the corresponding frequency was defined as the ripple frequency. Ripple energy was defined as the integral of values above 0.5 of maximal power in this frequency range. Tetrodes with a ripple energy <3 a. u. or with a frequency of SPW, carrying superimposed ripples, <0.2 Hz were deemed misplaced and excluded from further analysis.

Spike detection and sorting were performed using the Wave_clus program (Quiroga et al., 2004; Chaure et al., 2018) with parameters as shown in Extended Data Figure 1-1. The aim of the method is to attribute extracellular spikes to putative units based on the spike shape. Briefly, for spike detection the signal was bandpass filtered (300–3000 Hz) and thresholded based on the estimated standard deviation of the background noise. Then, wavelet transforms for the detected spikes were performed and a number of wavelet coefficients were automatically selected based on their deviation from normality. Based on these wavelet coefficients superparamagnetic clustering, a simulation of physical interactions of data points and their K-nearest neighbors, was performed, and the resulting clusters saved. Finally, these putative unit clusters were curated by the experimentalist in the Wave_clus GUI based on AP and autocorrelogram (AC) shape.

Units were further divided into putative PYRs and FSIs using four physiological parameters that have been shown to reliably distinguish these cell types (Sirota et al., 2008; English et al., 2017). For this, the K-Means algorithm was run on four *z* score normalized dimensions: log10 interspike interval (ISI) mode, log10 ISI median, burst percentage (defined as number of ISI <8 ms divided by number of all ISI × 100), and trough-to-right-peak latency. We expected at least three clusters, representing PYRs, FSIs, and at least one cluster containing other cell types. To find the optimal number of clusters, cluster quality was evaluated by the Calinski-Harabasz criterion. It exhibited a peak at 5 when testing between 3 and 15 clusters; thus, the number of clusters was set to 5 (Extended Data Fig. 1-2*A*,*B*). Subsequently, properties of clusters were used for the categorization of each cluster into PYR or FSI, respectively. One cluster exhibited the FSI phenotype (low ISI, high burst percentage); three clusters exhibited a PYR-like phenotype (slow firing, low burst percentage) and were merged; the remaining cluster displayed a partially bursting, partially slow-spiking phenotype, which could not be clearly classified as PYR or FSI and was assigned a third label (MIX). As both PYR and interneuron populations are known to be heterogenous, MIX likely represents various other cell classes including non-FSIs. As the focus of this study lies in the differentiation between PYR and FSI, the MIX group was excluded from the main analysis (Extended Data Fig. 1-2*E–L*).

Additionally, cumulative activity of all detected spikes in each recording was analyzed as multiunit activity (MUA).

The preferred ripple phase of a unit was defined as the 50th percentile of the unit-to-ripple-phase cross-correlogram. Firing precision was calculated from the width of the time window φ_in_ in °, within which the 25th to 75th percentiles of spikes on a ripple cycle occurs, as: phase specificity = (360° – 2 × φ_in_)/360°. Thus, phase specificity ranges from 0, in case of an equal distribution of spikes on the ripple cycle (φ_in_ = 180°), to 1, if all spikes occur precisely in phase (φ_in_ = 0°).

#### Patch clamp recordings

Following OGD, some slices were left for 20 min of recovery time in the interface-chamber (which was sufficient for SPW-R to re-emerge) while control slices were maintained in the interface-chamber without OGD, matching incubation time of OGD slices. Then, they were quickly transferred to a submerged-style, double-sided perfusion chamber (Hájos et al., 2009; Maier et al., 2009) for patch-clamp recordings (carbogen-saturated ACSF at 6 ml/min, 32–33°C). Under these conditions, all slices retained spontaneous SPW-R activity. Field potentials in the stratum pyramidale of CA1 were recorded simultaneously to the patch clamp recordings, using ACSF-filled glass microelectrodes (tip diameter, 2–4 μm). These signals were amplified 100×, low-pass filtered at 2 kHz, high-pass filtered at 0.3 Hz (EXT10–2FX amplifier; NPI Electronics) and digitized at 20 kHz.

#### Whole-cell voltage-clamp recordings

In slices obtained from WT animals CA1 pyramidal neurons were visualized with a DIR microscope. To reduce variability, we chose neurons with the typical soma shape in the superficial layer of proximal CA1 (60× magnification, BX51WI microscope, Olympus), as CA1 neurons have been shown to receive differential strengths of inhibition along the layers (Lee et al., 2014) and CA3–CA1 connectivity differs between the subregions of CA1 (Ishizuka et al., 1990). Whole-cell voltage-clamp recordings were performed with borosilicate electrodes (3–5 MΩ) filled with the following: 144 mm Cs-Gluc, 10 mm HEPES, 4 mm NaCl, 4 mm Mg-ATP, 0.3 mm Mg-GTP, and 10 mm phosphocreatine, pH 7.3, 1% Biocytin. Voltage signals were low-pass filtered at 8 kHz; current signals were low-pass filtered at 3 kHz (ELC-03XS amplifier; NPI Electronics) and both were digitized at 20 kHz. Pipette holding potential and offset were zeroed manually before seal formation. The liquid junction potential was calculated to be 15 mV and subtracted from the voltage data. Currents were recorded for 30 s each at −75-, −60-, 0-, and +15-mV nominal holding potential (corrected values: −90, −75, −15, and 0 mV). Access resistance and series resistance were monitored before and after each recording. Only cells with access resistance <30 MΩ and expressing a PYR-like electrophysiological phenotype were chosen; cells with an increase of series resistance >15% during the experiment were discarded.

Conductance changes during SPW-R events were analyzed through a custom MATLAB script based on a modification of the method by (Borg-Graham et al., 1998; Haider et al., 2006; Maier et al., 2016) to dissect inhibitory and excitatory conductance changes. Current traces (I_m_), recorded at different holding voltages (V_h_, listed above) during network events, were aligned by the peaks of individual SPW-R. Input conductance G_in_(t) was computed as the slope of the linear regression of the I–V relation between V_h_ and I_m_(t). The apparent reversal potential E_arev_ was taken from the intersection between I_m_(0) (during baseline before the SPW-R event) and I_m_(t) (during SPW-R-triggered postsynaptic events). The synaptic conductance change was calculated as G_chg_(t) = G_in_(t) − G_in_(0). AMPA and GABA reversal potentials were set to 0 mV (E_AMPA_) and −90 mV (E_GABA_), respectively. Inhibitory (G_chg, i_) and excitatory (G_chg, e_) conductance changes were then calculated as: G_chg, i_ = G_chg_ × (E_arev_ − E_AMPA_)/(E_GABA_ − E_AMPA_) and G_chg, e_ = G_chg_ − G_chg, i_.

For analysis of spontaneous postsynaptic currents (sPSC), −75- and +15-mV nominal holding voltage was used. After correction by the liquid junction potential these values correspond to 0 mV [E_rev_ for spontaneous EPSCs (sEPSCs)] and −90 mV (E_rev_ for sIPSCs with 4 mm intracellular and ∼130 mm extracellular [Cl^–^]). For analysis of these data, only SPW-R-free intervals were taken. Traces were bandpass-filtered (1–400 Hz for sEPSC; 1–200 Hz for sIPSC). A deconvolution-based algorithm was used to detect the PSCs. The deconvolution template comprised a double-exponential curve, resembling the physiological kinetics of recorded PSCs (Tauon of 1.25 ms, Tauoff of 3 ms for sEPSC; Tauon of 2.5 ms, Tauoff of 10 ms for sIPSC). PSCs were detected as local maxima in the deconvolved signal, combined with an amplitude threshold in the original signal as a secondary condition (>20 pA for sEPSC; >30 pA for sIPSC). Charge transfer of sPSC was defined as the integral within the time window of [sPSC peak – Tauon; sPSC peak + Tauoff]. Total sPSC current for the analysis time was defined as charge × frequency. I/E ratio was defined as sIPSC current/sEPSC current.

#### Cell-attached recordings

PV-IN in PV-tdTomato mice were visualized by epifluorescence (60× magnification, BX51WI microscope, Olympus). Borosilicate electrodes (3–5 MΩ) were filled with ACSF and cell-attached recordings were performed from CA1 PV-IN according to (Perkins, 2006). After achieving a loose seal (<1 GΩ) configuration, APs were monitored in voltage-clamp mode with the voltage manually set such that the measured holding current equaled zero. APs were then visible as brief deflections in the current trace. Throughout the article, we refer to this configuration as “cell-attached.” Current and LFP were recorded simultaneously for 200 s. A single threshold was used after offline bandpass filtering (100–5000 Hz) of recorded currents to determine spikes. Autocorrelations of spike times and their cross-correlations to LFP were calculated in MATLAB.

### Histology

Directly after recording, ∼1 h after HSD, slices were fixated in PFA for 12 h and thereafter stored in PBS at 4°C. Slices were then sub-sliced at 50 μm on a Leica V1200s vibratome (Leica Microsystems) and kept in a solution of v/v 31% ethylene glycol, 29% glycerin in PBS at −20°C until further processing. Slicing before experiments on living slices leads to damage and death of neurons close to the slice surface, and HI damage strongly correlates with depth within the tissue (Kann, 2012; Huchzermeyer et al., 2013). For these reasons, we chose two sub-slices from the middle portion of each original slice for staining.

We analyzed nuclear area and c-Fos co-expression by neuronal cell type. Sections were washed three times in PBS and blocked with 5% normal goat serum/0.3% Triton X-100 in PBS for 1 h. Thereafter, they were washed again in PBS and incubated with rabbit-anti-c-Fos (1:1000, Synaptic Systems), mouse-anti-PV (1:1000, Swant), and chicken-anti-NeuN (1:250, Synaptic Systems) primary antibodies in 1% normal goat serum/0.2% Triton X-100 in PBS for 18 h at room temperature. Upon washing with PBS, sections were incubated with Alexa Fluor 488 goat anti-rabbit (1:1000, Thermo Fisher Scientific), Cy3 goat anti-mouse (1:500, Dianova), Alexa Fluor 647 goat-anti-chicken (1:500, Thermo Fisher Scientific) secondary antibodies in 1% normal goat serum/0.2% Triton X-100 in PBS for 2 h at room temperature under light protection. Sections were then mounted onto slides with Fluoroshield (Sigma-Aldrich), containing DAPI, for nuclear staining. Fluorescence images were taken on a Nikon A1R (Nikon Instruments Inc.) confocal microscope system using a Nikon Plan Apo λ 20× NA 0.7 objective.

For image analysis, the experimentalist was blinded toward experimental conditions. Fluorescent images were preprocessed in Fiji ImageJ (Schindelin et al., 2012), as follows. First, regions of interest covering the stratum pyramidale and stratum oriens were selected for both CA1 and CA3, on which further processing and analysis were performed. Fluorescence channels were split into separate grayscale images, and the background was removed employing a rolling-ball algorithm. Then, cells and nuclei were segmented automatically using the cellpose algorithm (Stringer et al., 2021) with settings “pretrained model nuclei” for the DAPI and c-Fos channels, and “pretrained model cyto” for the PV and NeuN channels. Co-expression of c-Fos and nuclear size were determined automatically in Fiji ImageJ through a custom script.

### Statistical analyses

All statistical tests were performed in MATLAB. For statistical analyses of conductance changes, sPSC frequency and amplitude, and cell-attached spiking, we compared post-OGD to control groups. In the case of tetrode recordings, we performed within-group tests for the FSI and PYR groups, and for SPW-R parameters. Additionally, a between-group test for the relative frequency change of PYR compared with FSI was performed. The one-sample Kolmogorov–Smirnov test was used to test the normality of a given sample distribution. For normally distributed data, Student’s two-sided *t* test was used to test for statistical significance of differences between two groups, and Student’s paired sample *t* test for paired values. In case of non-normally distributed data, Wilcoxon’s rank-sum test and Wilcoxon’s signed-rank test were used for unpaired and paired data, respectively. For comparing the distributions of interevent intervals (IEIs) of sPSC between groups, the two-sample Kolmogorov–Smirnov test was used. For comparison of preferred ripple phase, the Watson–Williams test was used. The number n of experimental units, number of slices, and number of animals are indicated at the bottom of each figure legend. Null hypotheses were rejected at a significance level of α = 0.05. Throughout the article, data are presented as median [25th; 75th percentiles], if not stated otherwise.

## Results

### Neuronal activity is reduced strongest among FSIs after transient hypoxia

**Figure 1.**
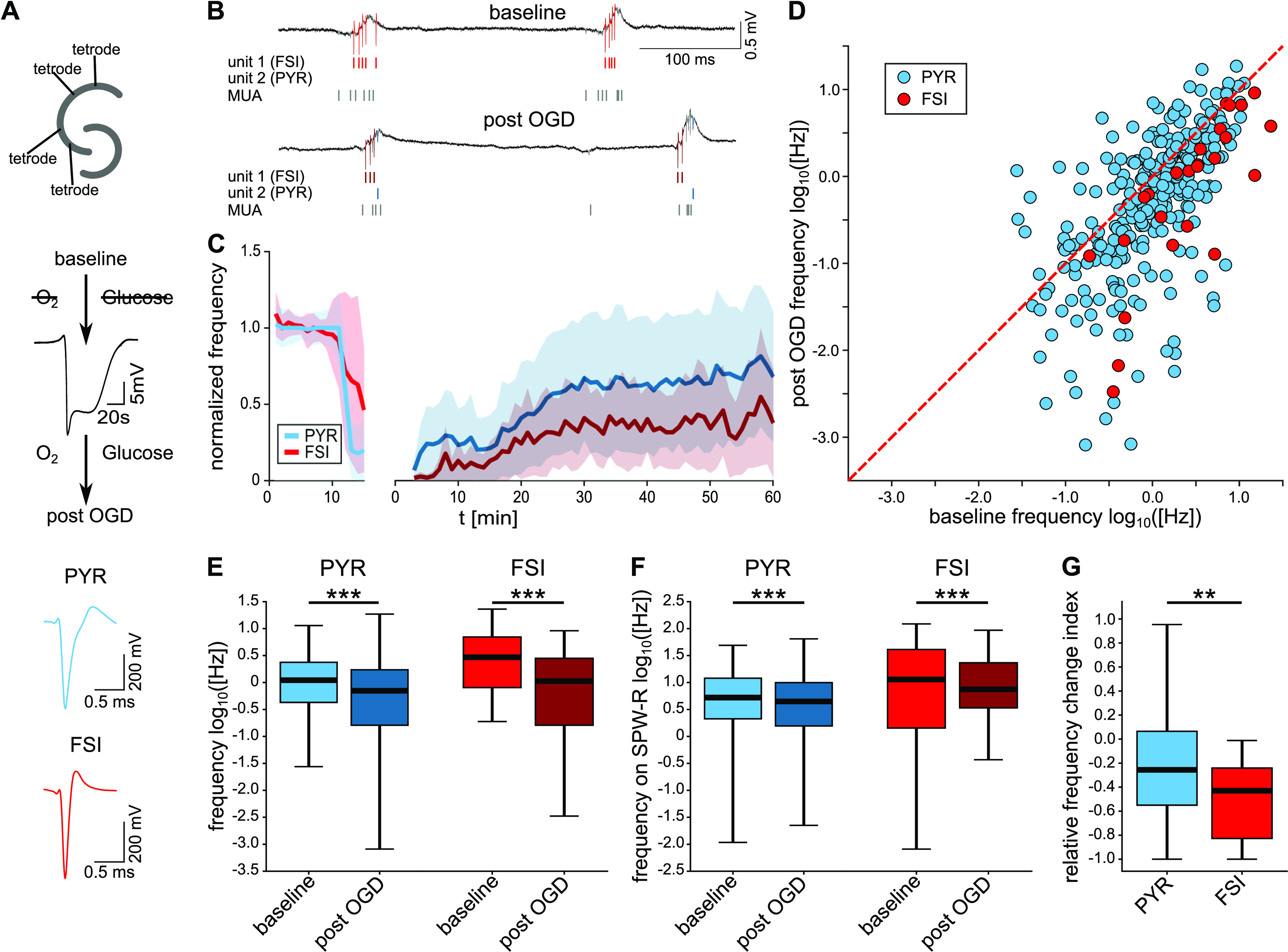
Firing of FSIs is reduced more strongly than PYR firing after OGD and recovery. ***A***, Experimental design. Top, Two tetrodes in each CA3 and CA1 regions of the hippocampal slice in an interface chamber. Middle, For OGD, perfusion was switched to an oxygen-glucose-free medium, until a HSD occurred (see representative LFP trace), whereupon perfusion was switched back to oxygenated ACSF. Bottom, Mean waveforms are shown for one putative FSI and one PYR. ***B***, Exemplary LFP recorded by tetrodes during baseline (top) and after recovery from OGD (bottom). Spike times of one FSI (red overlay), one PYR (blue overlay), and other units (MUA, gray overlay) are highlighted. ***C***, Binned median frequency of PYR (blue) and FSI (red), normalized by the median baseline frequency. Shaded regions represent the median absolute deviation. Minutes 0–10, baseline condition; minutes 10–15, OGD. Gap, HSD (because of variable latency from OGD onset to the occurrence of HSD in each experiment). Traces after gap, recovery during reperfusion. Time count on the right part of the *x*-axis begins with the time point of the HSD. ***D***, Logarithmic scatter plot of spiking frequencies of each unit, baseline (*x*-axis) compared with after 40–60 min of reperfusion (*y*-axis). Blue, PYR; red, FSI. ***E***, Unit spiking frequency during baseline and after recovery. *p*_PYR_ = 3 × 10^−8^. *p*_FSI_ = 8 × 10^−6^, Wilcoxon signed-rank test. ***F***, Frequency of unit spiking on SPW-R during baseline and after recovery. *p*_PYR_ = 0.0003. *p*_FSI_ = 0.0006, Wilcoxon signed-rank test. ***G***, Relative frequency change index, calculated as (f_post-OGD_ – f_baseline_)/(f_post-OGD_ + f_baseline_). *p* = 0.0028, Student’s *t* test. *n*_PYR_ = 296 units, *n*_FSI_ = 26 units from 25 slices, 12 mice; **p < 0.01, ***p < 0.001. Please refer to Extended Data Figure 1-1 for the parameters used in the spike sorting algorithm, Extended Data Figure 1-2 for the clustering of units into FSI and PYR, and Extended Data Figure 1-3 for the properties of the HSD.

We hypothesized that FSI activity might be more susceptible to hypoxia than that of PYRs. To test this, we measured unit activity from the hippocampal CA1 and CA3 regions with tetrodes before, during, and after transient OGD (Fig. 1*A*). Since FSI and PYR clearly differ in their respective firing pattern and AP waveform (Fig. 1*A*,*B*; Extended Data Fig. 1-2), we employed a clustering algorithm based on parameters reflecting these physiological properties (Extended Data Fig. 1-2) to differentiate between them. From 376 identified units, 26 units were classified as putative FSI and 296 units were clustered as putative PYR. The remaining 54 units did not fall into one of these classes and likely represent a mixed population of cells including bursting PYR and different types of interneurons (Klausberger and Somogyi, 2008).

10.1523/ENEURO.0221-21.2021.f1-1 Extended Data Figure 1-1 Parameters used in the Wave_clus algorithm. Download Figure 1-1, DOCX file.

**Figure 2.**
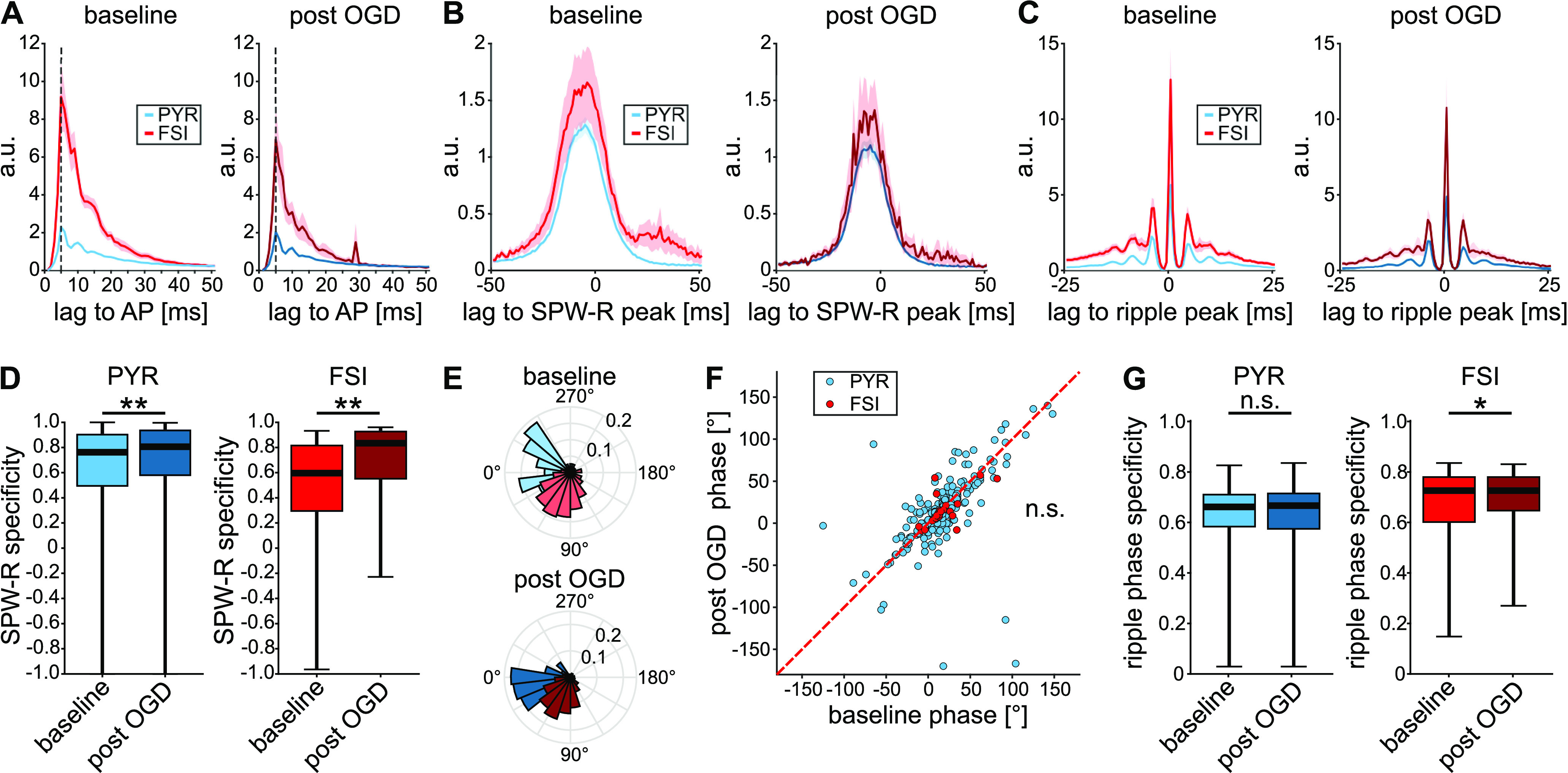
Coupling between unit firing and underlying network events recovers. ***A***, Mean autocorrelation of FSI (red) and PYR (blue) spike times. Dashed lines mark the first peak of the autocorrelation at 5 ms. ***B***, Mean cross-correlation between SPW-R peaks and FSI (red) or PYR (blue) spike times. ***C***, Mean cross-correlation between ripple peaks and FSI (red) or PYR (blue) spike times. In ***A–C***, shaded regions represent the SEM. Correlations were normalized, as follows: *n*_norm_ = *n*_corr_/sqrt(*n*_ev1_ × *n*_ev2_). The count of events *n*_corr_ within the displayed time window was divided by the square root of the product of the total occurrence of the event *n*_ev1_ to which the other event *n*_ev2_ was correlated, times the total occurrence of *n*_ev2_. ***D***, Specificity of spike times to SPW-R time frames, calculated as (f_inside_ – f_outside_)/(f_inside_ + f_outside_), where a value of 1 corresponds to firing being restricted to SPW-R and a value of −1 to firing only outside SPW-R. *p*_PYR_ = 0.0042. *p*_FSI_ = 0.0021, Wilcoxon signed-rank test. ***E***, Representative phase plot of spiking of one representative PYR (blue) and one FSI (red) unit on ripples, normalized by spike probability. 0° equals to the trough of the ripple cycle. ***F***, Scatter plot of preferred ripple phase for each unit, baseline paired with post-OGD values. Blue, PYR. Red, FSI. *p*_PYR_ = 0.904. *p*_FSI_ = 0.681, Watson–Williams test. ***G***, Specificity of spiking to the preferred ripple phase. *p*_PYR_ = 0.117. *p*_FSI_ = 0.012, Wilcoxon signed-rank test. *n*_PYR_ = 296 units, *n*_FSI_ = 26 units from 25 slices, 12 mice; *p < 0.05, **p < 0.01; n.s., not significant.

10.1523/ENEURO.0221-21.2021.f1-2 Extended Data Figure 1-2 Clustering of units by physiological properties. ***A***, t-Distributed stochastic neighbor embedding (t-SNE) 3D representation of units clustered by k-means across four physiological parameters, mode ISI, median ISI, burst percentage, and latency from AP trough to the right-hand peak. The k-means algorithm was set to five clusters (***B***). Based on physiological properties (***D***), three clusters were assigned the label PYR (high ISI, low burst-percentage), one cluster the label FSI (low ISI, high burst-percentage), and one cluster the label not clearly differentiable (high ISI, high burst-percentage; MIX). The three clusters representing putative PYR were merged. ***B***, Calinski–Harabasz values for between 3 and 15 clusters. Note the peak at five clusters. ***C***, ISI distribution of two representative units, clustered as a FSI and a PYR. Dotted lines mark median and mode of the distribution. ***D***, Histograms of the distribution of all units by clustering parameter. Top left, log_10_ of the ISI mode. Top right, log_10_ of the ISI median. Bottom left, Latency from AP trough to the right-hand peak. Bottom right, Percentage of spikes in bursts. ***E***, Logarithmic mode of the ISI. *p*_PYR, FSI_ < 1 × 10^−9^. *p*_PYR, MIX_ < 1 × 10^−9^. *p*_FSI, MIX_ = 0.44. ***F***, Logarithmic median of the ISI. *p*_PYR, FSI_ < 1 × 10^−9^. *p*_PYR, MIX_ = 0.73. *p*_FSI, MIX_ < 1* 10^−9^. ***G***, Latency from the trough to the right peak of the AP. *p*_PYR, FSI_ = 0.98. *p*_PYR, MIX_ = 4 × 10^−4^. *p*_FSI, MIX_ = 0.07. ***H***, Percentage of spikes in bursts. *p*_PYR, FSI_ < 1 × 10^−9^. *p*_PYR, MIX_ < 1 × 10^−9^. *p*_FSI, MIX_ < 1 × 10^−9^. ***I***, Overall AP frequency. *p*_PYR, FSI_ = 3 × 10^−9^. *p*_PYR, MIX_ = 0.16. *p*_FSI, MIX_ = 2 × 10^−9^. ***J***, Maximum slope of the AP during the repolarization phase. *p*_PYR, FSI_ = 0.01. *p*_PYR, MIX_ = 0.93. *p*_FSI, MIX_ = 0.02. ***K***, AP frequency during ripple oscillations. *p*_PYR, FSI_ < 1 × 10^−9^. *p*_PYR, MIX_ = 1 × 10^−5^. *p*_FSI, MIX_ = 6 × 10^−5^. ***L***, AP frequency during SPW-R events. *p*_PYR, FSI_ = 5 × 10^−8^. *p*_PYR, MIX_ = 0.22. *p*_FSI, MIX_ = 0.003. *n*_PYR_ = 296 units, *n*_FSI_ = 26, n_MIX_ = 54; *p < 0.05, **p < 0.01, ***p < 0.001; n.s., not significant. ANOVA followed by Tukey’s multiple comparison test. Download Figure 1-2, EPS file.

**Figure 3.**
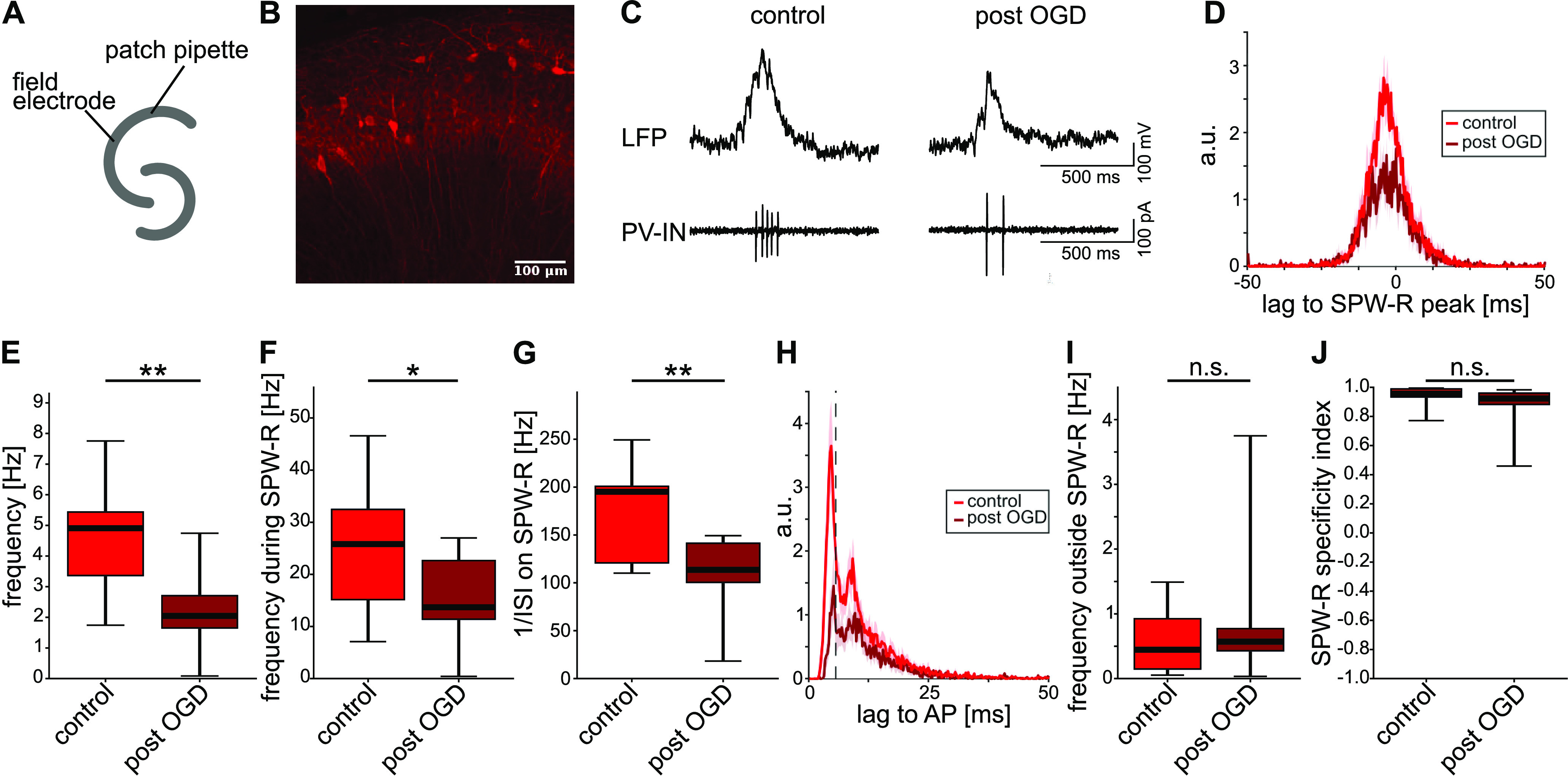
Cell-attached recordings reveal decreased spiking in CA1 PV-INs after transient OGD. ***A***, Experimental condition. A field electrode and a patch pipette were placed in the CA1 region of a hippocampal slice in a submerged chamber. ***B***, Representative image of the CA1 region in a horizontal slice from the PV-tdTomato mouse line at 20× magnification. ***C***, Representative traces. Top, SPW-R in the LFP. Bottom, PV-IN spikes recorded in the cell-attached voltage-clamp configuration simultaneously to the LFP recording. Left, Control recording. Right, Recording after OGD, HSD, and recovery. ***D***, Normalized mean cross-correlation between SPW-R peaks and spike times under control (red) and post-OGD (dark red) conditions. ***E***, Overall spike frequency. *p* = 0.0026. ***F***, Frequency during SPW-R-events. *p* = 0.046. ***G***, Instantaneous frequency (1/ISI) on SPW-R (if at least two spikes were detected during a SPW-R). *p* = 0.0043. ***H***, Normalized mean autocorrelation of spike times under control (red) and post-OGD (dark red) conditions. The dashed line marks the first peak of the autocorrelation at 5 ms. ***D***, ***H***, Shaded regions represent the SEM. Correlations were normalized, as follows: *n*_norm_ = *n*_corr_/sqrt(*n*_ev1_ × *n*_ev2_). ***I***, Frequency outside SPW-R. *p* = 0.476. ***J***, Specificity of spike times to SPW-R time frames, calculated as (f_inside_ – f_outside_)/(f_inside_ + f_outside_). *p* = 0.176. *n*_control_ = 12 cells from 5 slices and 3 mice, *n*_post-OGD_ = 10 cells from 4 slices and the same 3 mice; *p < 0.05, **p < 0.01; n.s., not significant. Student’s *t* test.

10.1523/ENEURO.0221-21.2021.f1-3 Extended Data Figure 1-3 Properties of HSD. ***A***, ***B***, Histograms displaying the amplitude (***A***) and duration (***B***) distribution of the negative DC shift characterizing the HSD; *n* = 10 events, 5 slices, 4 mice. ***C***, Survival plot of slices from the onset of OGD to the time of HSD; *n* = 25 slices, 12 animals. ***D–G***, Scatter plots and linear regressions (red lines) of the relation between the latency to HSD and the mean relative frequency (*f*) change index of FSIs (***D***; *n* = 14 slices), PYRs (***E***), CA1 SPW-Rs (***F***), and CA3 SPW-R (***G***) for each slice. *R*^2^ values are indicated at the bottom right of each plot. ***E–G***, *n* = 25 slices from 12 animals. Download Figure 1-3, EPS file.

Median baseline activity was 2.96 [0.81; 6.99] Hz for FSI and 1.10 [0.43; 2.37] Hz for PYR (Fig. 1*E*). In both groups, spiking was most pronounced during spontaneous SPW-R oscillations (Fig. 1*B*,*F*), consistent with previous findings (Both et al., 2008; Reichinnek et al., 2010). Switching to glucose-free and oxygen-free solution led to a spreading depolarization (SD) in all slices with delay times of 5.58 [4.25; 10.16] min (*n* = 25). The SD was characterized by a large negative deflection in the LFP, accompanied by a drastic reduction of unit activity in the LFP (Fig. 1*A*, representative trace; Extended Data Fig. 1-3*A–C*; please refer to Somjen, 2001; Dreier, 2011) for a review of SD mechanisms). Following reperfusion, most (>95%) of the previously identified units resumed activity, excluding acute cell death, as network events slowly re-emerged (Fig. 5*B–E*), reaching stable conditions 40–60 min after reperfusion (Fig. 1*C*,*D*).

FSI spiking showed a reduced recovery as compared with PYR throughout the whole time course of reperfusion (Fig. 1*C*, note difference between median recovery of FSI vs PYR). After 1 h, median activity of FSI reached only ∼36% of baseline values, in contrast to ∼64% in PYR (Fig. 1*C*). All putative FSI displayed lower firing rates after recovery from OGD, while the recovery of PYR was more diverse (Fig. 1*D*). Notably, some PYR regained higher activity rates than before OGD while all FSI showed values below the baseline situation. Still, median frequency was reduced significantly in both FSI and PYR (Fig. 1*E*). Firing during SPW-Rs was slightly reduced in both groups (Fig. 1*F*). Importantly, FSI frequency decreased significantly stronger than PYR frequency, as shown by the relative frequency change index, −0.43 [−0.83; −0.24] in FSI compared with −0.26 [−0.55; 0.07] in PYR (Fig. 1*G*, *p* = 0.003). Remarkably, these changes in unit firing did not correlate with the latency to HSD (Extended Data Fig. 1-3*D*,*E*).

### Coupling precision of units to SPW-R is preserved

Inhibitory interneurons play a critical role in the temporal organization of network activity. We therefore tested whether the observed effects of transient OGD disrupted the timing of neuronal discharges. Under baseline conditions, averaged, normalized spike ACs mirror the fast-spiking phenotype of FSI while PYR AC are of much lower amplitude, indicating that PYR fire less frequently during individual SPW-R events (Fig. 2*A*,*B*; for further analysis of FSI and PYR properties, refer to Extended Data Fig. 1-2*E–L*). Note that peaks in both FSI and PYR AC at ∼5 ms reflect the recorded ripple frequency (∼200 Hz). Interestingly, the overall shape of the AC appeared similar after recovery from OGD in both types of neurons, indicating preserved coupling to sharp waves and ripple oscillations (compare Fig. 2*A* and *C*). The specificity of FSI and PYR spiking during SPW-R periods, i.e., the relative sparseness of firing outside network events, increased slightly following OGD (Fig. 2*D*). Preferred ripple phases of firing for both FSI and PYR spikes did not change significantly (Fig. 2*E*,*F*). Similarly, firing precision, measured as ripple phase specificity (for more details, see Materials and Methods, Tetrode recordings) also remained high in both FSI and PYR (Fig. 2*G*). In summary, unit activity of FSIs and PYRs recovers its temporal coupling to SPW-R network activity after transient OGD.

### PV-INs display reduced spiking after recovery from OGD

To validate the findings from tetrode recordings, we performed cell-attached recordings from CA1 PV-INs together with LFP recordings (Fig. 3*A–C*). Measurements were performed in a submerged chamber on five (control) and four (post-OGD) slices from the same three mice where PV-IN could be identified by their tdTomato fluorescence (Fig. 3*A*,*B*; see Materials and Methods, Cell-attached recordings). We compared slices after OGD/HSD and recovery with controls incubated in normal ACSF.

Following transfer from the interface-type to the submerged-type chamber, all slices from both the control and post-OGD group displayed spontaneous SPW-R events in the LFP (Fig. 3*C*, representative trace). In cell-attached recordings all PV-IN fired spontaneous APs which were strongly coupled to SPW-R (Fig. 3*D*). In line with the findings from tetrode recordings, the median spiking frequency of PV-IN in the post-OGD group was ∼60% lower than that of controls (2.05 [1.66; 2.71] Hz compared with 4.91 [3.37; 5.44] Hz; Fig. 3*E*, *p* = 0.003). Additionally, OGD-challenged PV-IN fired less APs per SPW-R than controls (Fig. 3*F*), consistent with the higher ISI (Fig. 3*G*). Nevertheless, both the OGD and the control group displayed peaks at ∼5 ms in the averaged AC (Fig. 3*H*), corresponding to the ripple frequency and indicating preserved coupling to individual ripple cycles. Ripple oscillations in the LFP signal were, however, less pronounced under these experimental conditions, such that a cross-correlation analysis did not yield clear results. Firing outside of SPW-R remained low in the OGD group (Fig. 3*I*) and PV-IN spiked almost exclusively on SPW-R events, as shown by a specificity index close to 1 in both groups (Fig. 3*J*). Thus, cell-attached recorded PV-IN show a strong reduction of spiking frequency, yet preserved spike coupling to SPW-R, validating the findings from tetrode recordings.

### Postsynaptic conductance changes during SPW-R are reduced

**Figure 4.**
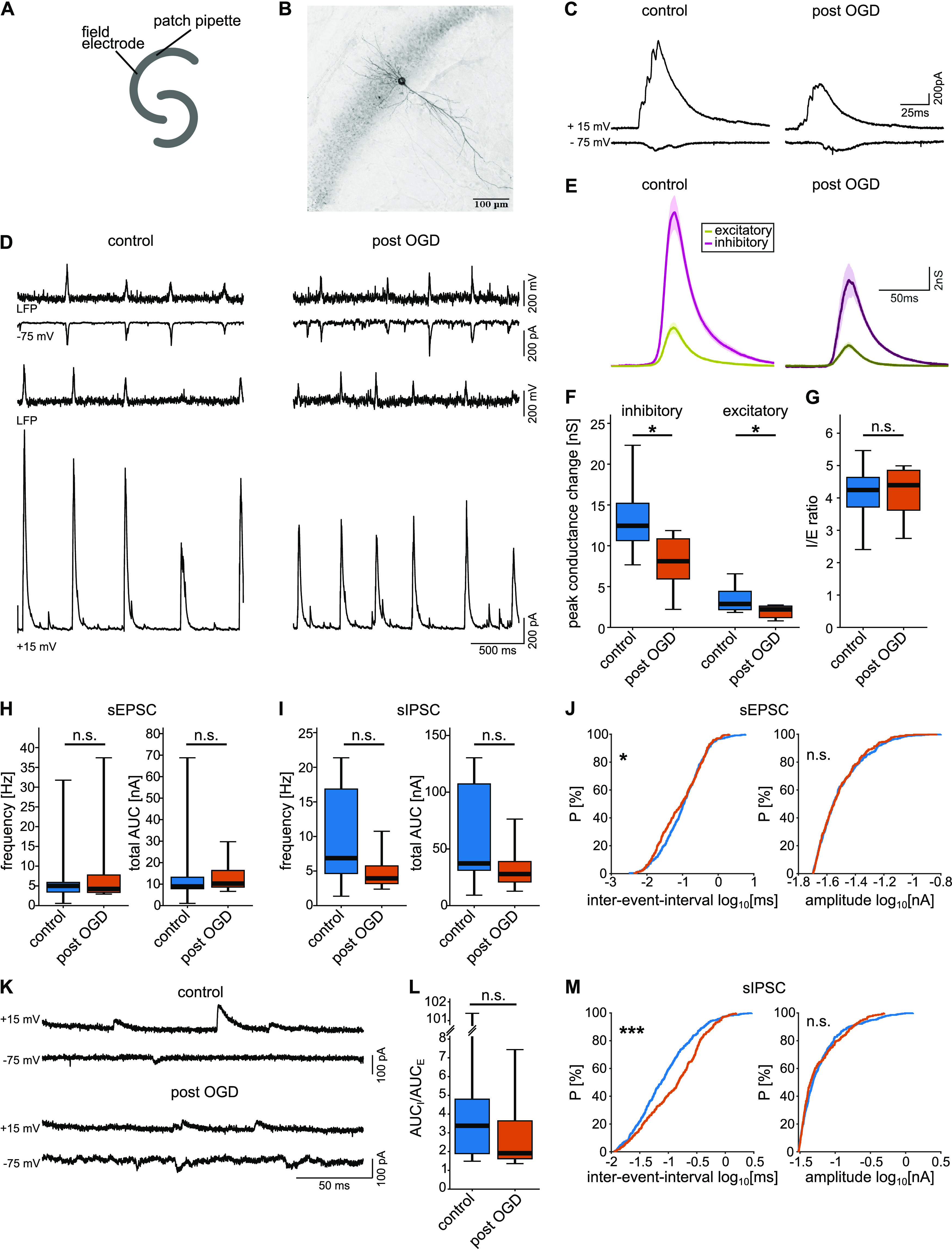
Whole-cell voltage-clamp recordings from CA1 PYRs yield reduced conductance changes, yet preserved network balance after OGD. ***A***, Experimental design. A field electrode and a patch pipette placed in the CA1 region of the hippocampal slice, recorded in a double-perfused, submerged-style chamber. ***B***, Summed view of a representative 60× confocal image z-stack of a biocytin-filled CA1 PYR from the post-OGD group. The background was removed using the rolling-ball algorithm and the LUT was inverted. ***C***, Representative current traces during SPW-R, magnified from ***D***, at 15- and −75-mV nominal holding voltage. ***D***, Representative traces of LFP and voltage-clamp recordings at 15- and −75-mV nominal holding voltage. ***E***, Mean conductance changes during SPW-R, calculated by the Borg-Graham method (for details, see Materials and Methods). Shaded regions represent the SEM. Purple, inhibitory conductance. Khaki, excitatory conductance. ***F***, Peak conductance changes during SPW**-**R. *p*_inhibitory_ = 0.0202, *p*_excitatory_ = 0.0341, Student’s *t* test. ***G***, I/E ratio, calculated as peak inhibitory conductance changes divided by peak excitatory conductance changes. *p* = 0.9835, Student’s *t* test. ***H***, sEPSCs outside of SPW-Rs. Left, Frequency of sEPSCs under control (blue) or post-OGD (orange) condition. *p* = 0.7076, Student’s *t* test. Right, Total AUC, calculated as the area of the mean synaptic current curves multiplied by the frequency. *p* = 0.6574, Student’s *t* test. ***I***, sIPSCs outside of SPW-Rs. Left, Frequency of sIPSCs. *p* = 0.0875, Student’s *t* test. Right, Total AUC. *p* = 0.1355, Student’s *t* test. ***J***, For the cumulative distribution of sEPSC IEIs and sEPSC amplitudes, 50 data points from each cell were pooled together. Left, Cumulative distribution of sEPSC IEI. *p* = 0.027, two-sample Kolmogorov–Smirnov (KS) test. Right, Cumulative distribution of sEPSC amplitude. *p* = 0.946, two-sample KS test. ***K***, Magnification of current traces in ***D***, displaying sPSCs outside SPW-R. Nominal holding potential is displayed. ***L***, I/E ratio of sPSC outside SPW-R was computed as AUC_I_/AUC_E_. *p* = 0.3148, Wilcoxon rank-sum test. ***M***, For the cumulative distribution of sIPSC IEI and sIPSC amplitudes, 50 data points from each cell were pooled together. Left, Cumulative distribution of sIPSC IEI. *p* = 4 × 10^−9^, two-sample KS test. Right, Cumulative distribution of sIPSC amplitude. *p* = 0.061, two-sample KS test. *n*_control_ = 10 cells from 7 slices and 5 mice. *n*_post-OGD_ = 7 cells from 5 slices and the same 5 mice; *p < 0.05, ***p < 0.001; n.s., not significant.

To test whether the observed preferential decrease in FSI spiking led to changes in I/E balance, we performed whole-cell patch clamp recordings from CA1 PYRs in both post-OGD and control slices (Fig. 4*A*,*B*,*D*). We recorded PSCs at different holding potentials from −75 to +15 mV and calculated inhibitory and excitatory conductance changes during SPW-R, using the method previously described (Borg-Graham et al., 1998; Fig. 4*C–G*). In line with previous studies, we observed that input to CA1 PYRs during SPW-R consisted predominantly of a large inhibitory conductance increase and a 4- to 5-fold smaller excitatory component (Fig. 4*E*).

Input resistance was similar in control and post-OGD neurons, 139.83 [129.19; 155.04] MΩ in the control group compared with 162.83 [118.72; 186.97] MΩ in the post-OGD group (*p* = 0.4121).

The amplitude of the inhibitory conductance changes during SPW-R was significantly smaller in the post-OGD group (8.1 [5.9; 10.8] nS) compared with the control group (12.5 [10.6; 15.2] nS; Fig. 4*F*; *p* = 0.02). Likewise, excitatory conductance changes during SPW-R were smaller in slices after OGD (2.2 [1.2; 2.6] nS) than in slices from control conditions (2.9 [2.2; 4.4] nS; Fig. 4*F*; *p* = 0.03). These similar changes resulted in unaltered ratios between inhibitory and excitatory conductance changes during SPW-R network events (Fig. 4*G*).

We furthermore evaluated sPSCs measured in-between SPW-R network events (Fig. 4*H–M*). Frequency and area under the curve (AUC) of inhibitory and excitatory sPSCs were not significantly altered after transient hypoxia (Fig. 4*H*,*I*), despite an apparent trend toward decreased median sIPSC frequency. Cumulative distributions of IEIs displayed a significant shift of IEIs for sIPSC toward larger intervals, while sEPSC IEI were slightly shifted to shorter intervals (Fig. 4*J*,*M*). Amplitude distribution was unaltered between the two conditions for both sIPSC and sEPSC (Fig. 4*J*,*M*). Similar to synaptic conductances during SPW-R, the ratio of inhibitory to excitatory AUC outside SPW-R was not significantly reduced, despite some apparent trend to a relative decrease in inhibition (Fig. 4*L*).

Overall, following the recovery from OGD, no significant change in I/E ratio during or outside SPW-R was observed.

### Network events recover partially

**Figure 5.**
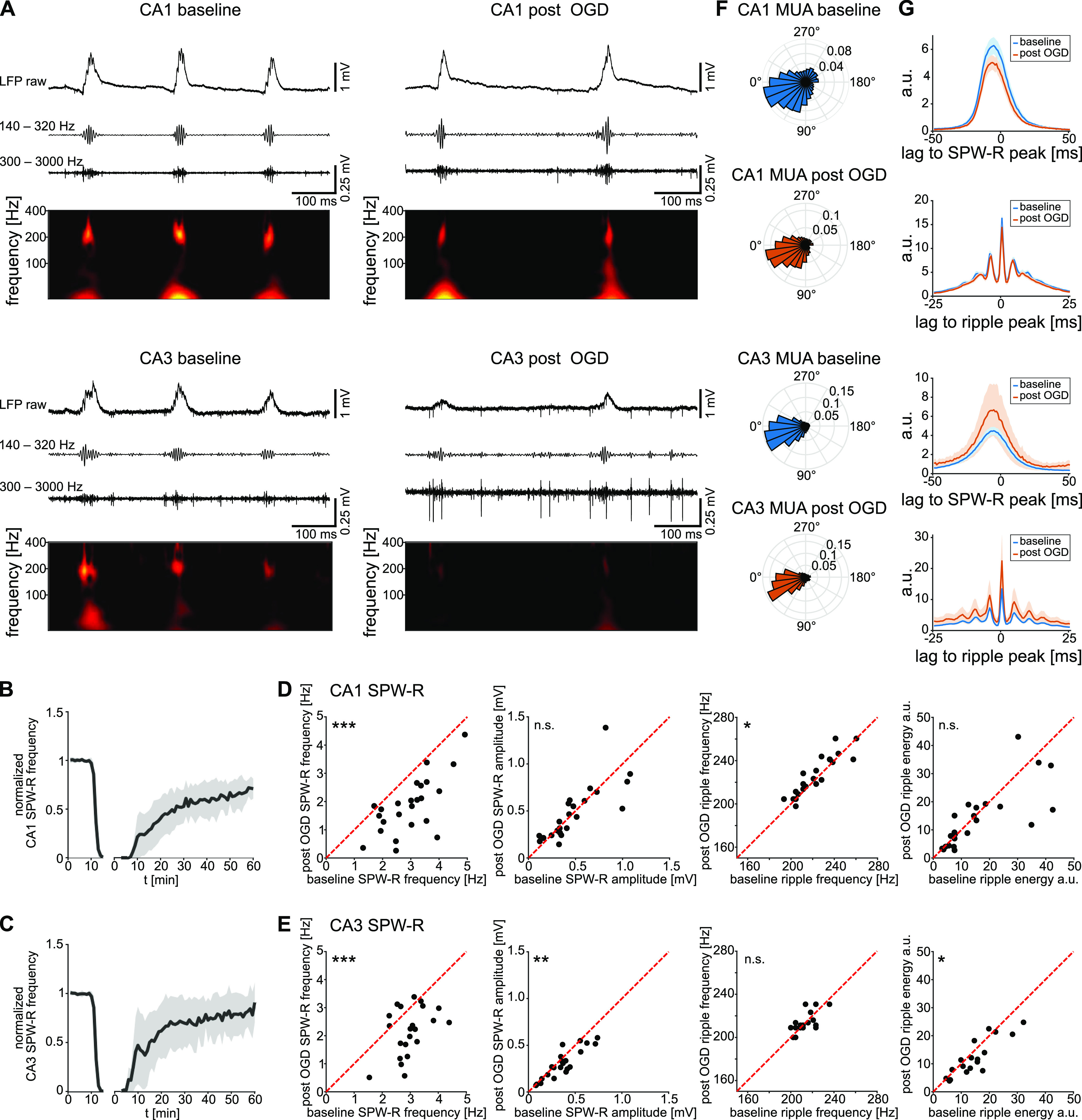
SPW-Rs re-emerge with reduced incidence and altered ripple properties. ***A***, Representative LFP traces, exhibiting spontaneous SPW-R activity, recorded simultaneously from CA1 (top) and CA3 (bottom). Displayed traces, under baseline conditions (left) and after OGD (right) stem from the same recording. Below each raw trace, the corresponding bandpass filtered traces to highlight ripples (140–320 Hz) and MUA (300–3000 Hz), and the power-spectrogram are displayed. ***B***, ***C***, Binned median amplitude of SPW-R (***B***, CA1; ***C***, CA3), normalized by the median baseline amplitude. Shaded regions represent median absolute deviation. Minutes 0–10, baseline condition; minutes 10–15, OGD. Gap, HSD. Traces after gap, recovery during reperfusion. Time count on the right part of the *x*-axis begins with the time point of the HSD. ***D***, ***E***, SPW-R characteristics (***D***, CA1; ***E***, CA3). From right to left, SPW-R incidence, *p*_CA1_ = 3 × 10^−7^, *p*_CA3_ = 0.0001; SPW-R amplitude, *p*_CA1_ = 0.9624, *p*_CA3_ = 0.0024; ripple frequency, *p*_CA1_ = 0.0465, *p*_CA3_ = 0.8371; ripple energy, *p*_CA1_ = 0.2403, *p*_CA3_ = 0.0112. Student’s paired *t* test. ***F***, Phase plot of MUA on ripples from a representative recording, normalized by spike probability. ***G***, Mean cross-correlation between SPW-R peaks and MUA spikes (first and third graph), and mean cross-correlation between ripple peaks and MUA spikes (second and fourth graphs) during baseline (blue) and post-OGD (orange) conditions. Top two graphs, CA1. Bottom two graphs, CA3. Shaded regions represent the SEM. Correlations were normalized, as follows: *n*_norm_ = *n*_corr_/sqrt(*n*_ev1_ × *n*_ev2_). *n*_CA1_ = 24 slices from 12 mice, *n*_CA3_ = 22 slices from 12 mice; *p < 0.05, **p < 0.01, ***p < 0.001; n.s., not significant.

Fast, rhythmic inhibition is necessary for coordinated network activity. We hypothesized that deficits in fast inhibitory signaling might be accompanied by altered ripple oscillations. We therefore evaluated waveform parameters of extracellularly recorded SPW-R before, during, and after transient OGD (Fig. 5*A*).

In all slices, the LFP displayed spontaneous SPW-R activity, consisting of the slow, large amplitude sharp wave occurring with an incidence of ∼3 Hz and fast superimposed ripple oscillations (∼220 Hz). OGD abolished SPW-R activity within 3–5 min. Following OGD, HSD, and re-oxygenation, SPW-R events re-emerged in all slices. Amplitude and frequency of the recovered events stabilized ∼40 min after HSD (Fig. 5*B*,*C*). We compared SPW-R 40–60 min after HSD to baseline SPW-R.

Following OGD, a number of differences to baseline SPW-R were apparent. First, the incidence of SPW-R events was reduced from 3.03 [2.25; 3.56] Hz to 1.84 [1.23; 2.47] Hz in the CA1 region and from 2.94 [2.65; 3.24] Hz to 2.30 [1.69; 2.98] Hz in the CA3 region (Fig. 5*D*,*E*). In both regions, SPW-R incidence did not correlate with the latency to HSD (Extended Data Fig. 1-3*F*,*G*). Second, SPW-R amplitude was reduced in CA3 while it stayed unaltered in CA1 (Fig. 5*D*,*E*). Third, ripple parameters also displayed treatment-dependent and region-dependent differences. The frequency of ripple oscillations was increased in the CA1 region post-OGD, while it remained mostly unchanged in the CA3 region. Ripple energy was lower in CA3 with no clear change in the CA1 region (Fig. 5*D*,*E*).

To evaluate the coupling of unbiased, cumulative neural activity to SPW-R, we compared MUA during baseline and after recovery. As expected, baseline MUA was strongly coupled to SPW-R events in both CA1 and CA3 (Fig. 5*F*,*G*). On ripple oscillations, MUA took place preferentially during the trough (0–30°) of ripple cycles, leading to an oscillatory shape of the averaged cross-correlogram (Fig. 5*F*,*G*). Following OGD, this characteristic coupling to SPW-R oscillations remained largely unaltered (Fig. 5*F*,*G*). Thus, slices appear to display deficits in the initiation of sharp waves and ripple-oscillations following transient OGD, while the characteristic SPW-R waveform and the entrainment of neuronal activity are mostly preserved.

### Neurons are activated differentially by transient hypoxia depending on hippocampal subregion

**Figure 6.**
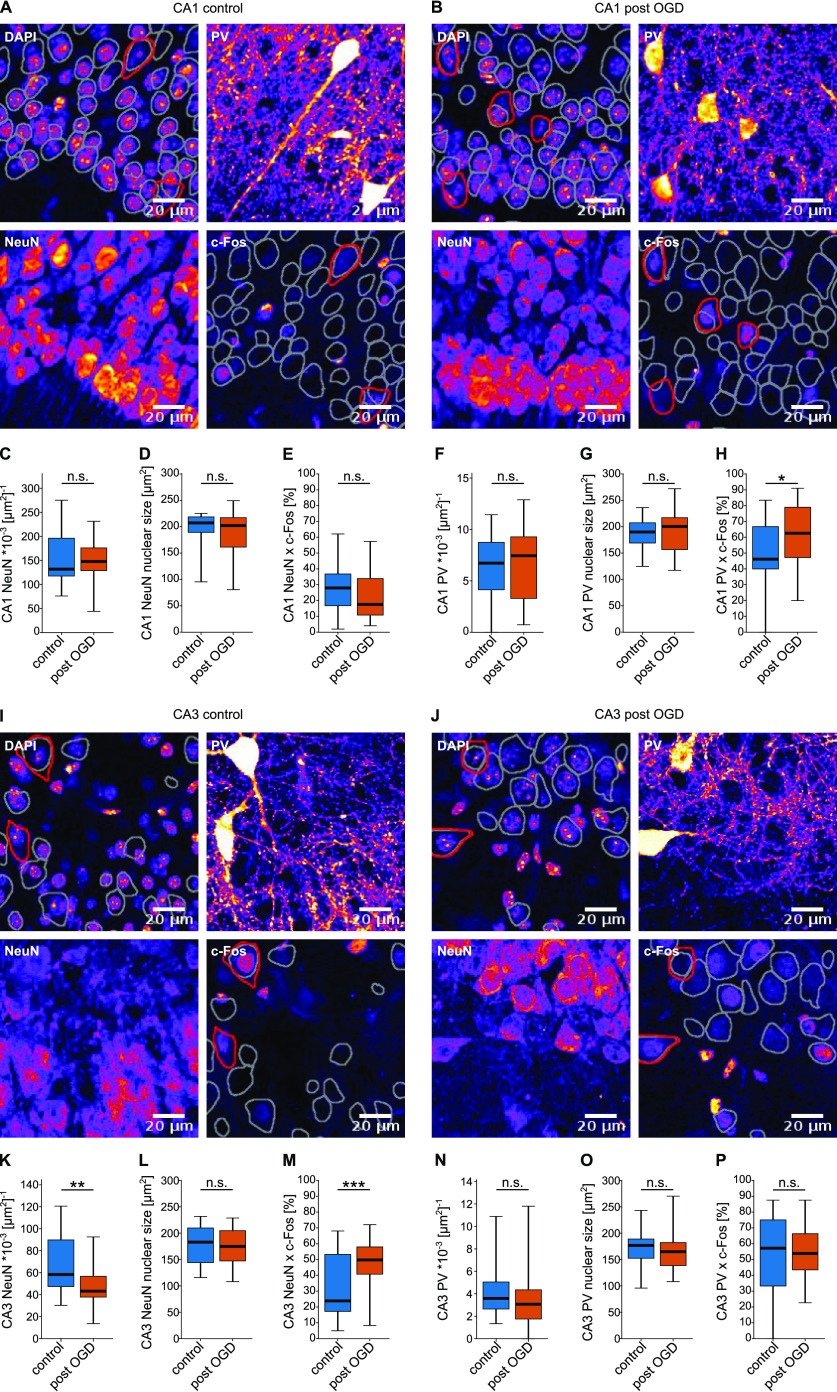
Altered c-Fos expression after OGD depends on region and cell type. ***A***, ***B***, ***I***, ***J***, Fire-LUT of representative four-channel confocal images of the CA1 (***A***, ***B***) and CA3 (***I***, ***J***) region in quadrupel-stained hippocampal slices at 20× magnification after background removal with the rolling-ball algorithm. Channels are indicated in the top left corner of each image. In the c-Fos and DAPI channel gray and red overlays denote NeuN+ and PV+ cell outlines as determined by the cellpose algorithm, respectively. ***C***, Number of NeuN+ cells per 1000 μm^2^ in CA1. *p* = 0.5846. ***D***, Nuclear area of NeuN+ neurons in CA1. *p* = 0.4262. ***E***, Percentage of NeuN+/PV– neurons co-expressing c-Fos in CA1. *p* = 0.0995. ***F***, Number of PV+ cells per 1000 μm^2^ in CA1. *p* = 0.5702. ***G***, Nuclear area of PV+ neurons in CA1. *p* = 0.833. ***H***, Percentage of PV+ neurons co-expressing c-Fos in CA1. *p* = 0.0305, Student’s *t* test. ***K***, Number of NeuN+ cells per 1000 μm^2^ in CA3. *n*_control_ = 26, *n*_post-OGD_ = 34. *p* = 0.0016. ***L***, Nuclear area of NeuN+ neurons in CA3. *p* = 0.6601. ***M***, Percentage of NeuN+/PV– neurons co-expressing c-Fos in CA3. *p* = 0.0007. ***N***, Number of PV+ cells per 1000 μm^2^ in CA3. *n*_control_ = 26, *n*_post-OGD_ = 34. *p* = 0.1363. ***O***, Nuclear area of PV+ neurons in CA3. *p* = 0.5933. ***P***, Percentage of PV+ neurons co-expressing c-Fos in CA3. *p* = 0.7764. *n*_control_ = 26 slices from 7 animals, *n*_post-OGD_ = 34 slices from 7 animals (for cell marker counts). *n*_CA1 control_ = 25 slices from 7 animals, *n*_CA1 post-OGD_ = 28 slices from 7 animals, *n*_CA3 control_ = 22 slices from 7 animals, *n*_CA3 post-OGD_ = 23 slices from 7 animals (for DAPI and c-Fos co-expression, slices with less than five PV-IN in the ROI were excluded); *p < 0.05, **p < 0.01, ***p < 0.001; n.s., not significant. Student’s *t* test.

So far, we observed a differential effect of transient hypoxia on electrophysiologically measured activity in different hippocampal regions and neuronal types. To further elucidate the effects of transient OGD on neuronal activation, we tested for cell death and neuronal activation across PYR and PV-IN in both CA1 and CA3. Most of the common apoptosis and stress markers reach their maximal expression within several hours after the insult, which is beyond the time window of our *in vitro* experiment. Acute cell death, however, is readily observable within short time intervals after an insult, using nuclear cross section area as a morphologic marker (Bonde et al., 2002). Additionally, co-expression of the immediate-early gene product c-Fos, known to be upregulated after hypoxic insults and spreading depression (Dragunow et al., 1990; Kiessling et al., 1993), was quantified by immunofluorescence. To these ends we performed quadruple stainings for c-Fos, PV, NeuN, and DAPI in slices that had undergone OGD and compared them to slices after control incubation (Fig. 6*A*,*B*,*I*,*J*).

There was no difference in the number of CA1 NeuN+/PV– cells or CA1 PV+ cells per area between conditions (Fig. 6*C*,*F*). The number of CA3 NeuN+/PV– cells, however, was decreased post-OGD (43.3 [37.9; 56.8] × 10^−3^ [μm^2^]^−1^ compared with 58.4 [47.5; 89.8] × 10^−3^ [μm^2^]^−1^; Fig. 6*K*), while the number of CA3 PV+ cells was unchanged (Fig. 6*N*). Neither NeuN+ nor PV+ neurons showed clear signs of necrosis after transient OGD, as the nuclear area was unchanged (Fig. 6*D*,*G*,*L*,*O*). This was the case in both the CA1 and CA3 region.

c-Fos staining revealed a differential effect of transient OGD on cellular activation. In the CA1 region the percentage of c-Fos-positive cells was increased among PV-IN from 46.2% [40.0%; 66.7%] to 62.5% [47.2%; 78.9%] (*p* = 0.03) after transient OGD while it was unchanged for NeuN+/PV– cells (Fig. 6*E*,*H*). In the CA3 region, on the other hand, the percentage of c-Fos-positive NeuN+/PV– neurons was significantly higher in slices which had undergone transient hypoxia than in controls (49.8% [40.7%; 57.9%] compared with 23.9% [17.3%; 53.2%]; *p* = 0.0007) with no change among PV-INs (Fig. 6*M*,*P*). Thus, a moderate and differentiated effect of transient OGD on c-Fos protein expression was visible.

## Discussion

While cascades leading to acute necrosis and apoptosis after a severe insult are relatively well understood, knowledge about functional recovery after transient HI is still rather vague. Our data show a reduced recovery of electrophysiologal FSI activity compared with PYR combined with alterations of synchronous network events.

The vulnerability of PV+ FSI is a particularly important issue, and it is presently unclear to which extent their altered function contributes to impaired posthypoxic network activity (Johansen et al., 1983; Nitsch et al., 1989; Moga et al., 2002; Wang, 2003; Povysheva et al., 2019; Elzoheiry et al., 2020). Earlier studies found unchanged numbers of PV-IN in contrast to reduced CA1 PYR numbers several days after HI *in vivo* (Johansen et al., 1983; Nitsch et al., 1989). Calcium-binding properties of PV are discussed to reduce neurotoxic effects of calcium overload during HI (Tortosa and Ferrer, 1993). However, more recent studies indicate impaired function of inhibitory neurons compared with excitatory neurons after transient metabolic insults (Moga et al., 2002; Wang, 2003; Povysheva et al., 2019; Elzoheiry et al., 2020). It is important to note the methodical differences in those studies in terms of insult severity and outcome measures (histologic cell death vs electrophysiological function).

Our data from tetrode recordings in acute hippocampal slices are in line with those more recent reports (Povysheva et al., 2019; Elzoheiry et al., 2020), demonstrating an increased functional vulnerability of FSI compared with PYR. However, while Povysheva et al. (2019) found that PV-IN held in current-clamp during transient OGD showed no recovery of membrane potential and spiking activity, which the authors interpreted as a sign of cell death, cells remained clearly viable under our experimental conditions, but showed distinct functional deficits. In contrast to results from Elzoheiry et al. (2020), who employed a pharmacological metabolic challenge, we did not observe an overall increase in PYR activity, in line with the unaltered I/E ratio in our cellular recordings.

Results from tetrode-recorded putative FSI are consistent with data from cell-attached recordings from PV-IN in CA1, both showing a reduction of overall spiking frequency of ∼60%. While excitatory feedback from PYR to PV-IN drives their activity and stabilizes of neuronal ensembles (Cornford et al., 2019), this result cannot be explained by diminished excitatory drive alone as the reduction of PV-IN spiking by far exceeds the reduction of PYR activity. Several implications may arise from this reduced FSI activity: (1) a relative lack of inhibition is expected to lead to decreased I/E ratios, which may even result in increased activity of PYRs and, in the extreme case, epileptic seizures (Stief et al., 2007); (2) a loss of precisely timed phasic inhibition by FSI might impair temporal cell-to-network coupling and thereby reduce ripple-associated spike timing (Gan et al., 2017); and (3) altered reciprocal interaction between FSI and PYR could affect the generation of SPW-R activity (Schlingloff et al., 2014).

OGD led to similarly decreased inhibitory and excitatory conductance changes in CA1 PYR during SPW-R. As a result, I/E ratio during SPW-R remained largely unaltered. This finding seems to be at odds with the observed stronger reduction in FSI firing frequency as compared with PYR. One possible explanation would be that a sufficiently high I/E ratio may be a precondition for the occurrence of SPW-R (Hájos et al., 2013; Schlingloff et al., 2014), such that SPW-Rs occur less frequently, but, once generated, show a near-normal I/E value. We therefore examined sPSCs in-between SPW-R. Here, sIPSCs, but not sEPSCs, exhibited increased IEI. This would suggest reduced inhibition; the I/E ratio, however, was not significantly altered. Thus, subtle differences in neuronal behavior can be observed depending on network state after recovery from OGD, while the relative deficiency in fast inhibition is not accompanied by a drastic increase of the excitatory drive onto PYR. This is relevant, as it largely excludes a potentiation of excitotoxic processes triggered by HI during the acute recovery phase.

Additionally, in none of our experiments epileptiform or ictal-like events occurred, showing that in our relatively mild HI model a sufficient level of tonic inhibition remains after OGD. The insult severity in our study thus rather resembles the relatively mild transient ischemic attacks, which are far less linked to epilepsy than a full stroke (Arntz et al., 2013).

Homeostatic mechanisms may compensate for the lower frequency of discharges in PV-IN (Turrigiano et al., 1998). This may include increased quantal content of IPSCs, in line with the largely unaltered amplitude of spontaneous inhibitory currents, or even changes in quantal size (Hartmann et al., 2008), which were not measured in the present study. Also, non-PV interneurons innervating PYR may partially compensate for the loss of activity on PV-IN, such that the overall balance between excitation and inhibition was preserved, although PV-IN constitute the class thought to be most relevant to SPW-R (Klausberger and Somogyi, 2008). As a result, we did not observe overt hyperexcitability of PYR.

In contrast, PYR firing frequency was reduced and their temporal coupling to the network was preserved. Of course, further mechanisms may contribute to the behavior of PYR post-OGD. Increased firing may result from the reduction of inhibition (Elzoheiry et al., 2020), increased excitability (Fan et al., 2008), and ischemic long-term potentiation (Maggio et al., 2015). On the other hand, factors like the lack of energy-producing metabolites following hypoxia (Kass and Lipton, 1989), perturbed neuronal ion homeostasis (Müller and Somjen, 2000), and calcium-triggered disruption of the axon-initial segment morphology (Revah et al., 2019) may reduce neuronal activity following OGD and HSD.

As both FSI and PYR activity is entrained by SPW-R oscillations, network interactions have a major influence on the results of this study. Future studies with other experimental designs are required to investigate intrinsic factors of FSI vulnerability and their functional outcome in the context of other network states.

As has been shown previously (Hefter et al., 2016), SPW-Rs were transiently abolished during OGD, but re-emerged shortly after reperfusion. Spontaneous network oscillations partially recovered in all slices and their overall waveform, consisting of slow large-amplitude sharp waves and superimposed high-frequency ripples, was maintained. However, several subtle, region-specific changes could be observed. Reduced incidence of SPW-R in both CA3 and CA1 indicates impaired generation of oscillatory activity following OGD. While the exact mechanism of SPW-R generation is still debated, activation of FSI by PYR is a critical part. It has been shown that FSI firing is associated with succeeding SPW-R (Bazelot et al., 2016). During SPW-R, hippocampal neurons repeatedly fire in specific patterns and on a certain phase of the ripple cycle (Reichinnek et al., 2010). This replay is thought to be pivotal for consolidation of episodic and special memory in the hippocampus (van de Ven et al., 2016). As discussed above, reduced SPW-R oscillations may hint toward impaired interplay between FSI and PYRs.

In contrast to CA1, SPW-R amplitude and ripple energy were reduced in CA3 after reperfusion, indicating that either less CA3 neurons or smaller PSCs contribute to the LFP signature of these oscillations after OGD. On the other hand, these smaller CA3 SPW-R still seem to be sufficient to elicit SPW-R of unaltered magnitude in CA1. These networks appear to be less affected by OGD than those in CA3, possibly because of reduced inhibitory control or to hypoxia-triggered plastic effects such as ischemic LTP (Crepel et al., 2003). A subtle increase in ripple frequency can be observed in CA1, possibly indicating a smaller ensemble size contributing to these oscillations. Intact hippocampo-neocortical connectivity is considered crucial for memory and executive function (Girardeau et al., 2009; Sigurdsson and Duvarci, 2016). Reduced output of SPW-R from CA1 might impair these connections and thus cognitive function.

PV-IN firing is strongly coupled to SPW-R and reaches frequencies around 200 Hz in cell-attached recording. Notably, the observed reduction of PV-IN frequency mostly results from reduced firing on SPW-R, while spiking outside of SPW-R is unaltered. The reduction of phasic inhibition seems to be remarkably well compensated, having quite little impact on the precisely timed network function.

Our histologic data largely exclude that the observed functional alterations are explained by cell death, as the absence of nuclear shrinkage in our data indicates that no significant amount of cell death occurred (Bonde et al., 2002). Nuclear shrinkage could be a sign of either beginning apoptosis or necrosis, while most common markers of these processes are not upregulated at the short time scale of the experiment. The apparent absence of dying cells was expected because of the short duration of the OGD insult. This notion is also in line with the fact that most (>95%) identified units re-appeared after the OGD (Fig. 1*D*). However, it cannot be excluded that cell death would have occurred beyond our experimental time window of ∼1 h after OGD. Indeed, hippocampal neurons are known to undergo delayed apoptosis 24–72 h after HI (Kirino, 2000). The surprising reduction in CA3 NeuN+/PV– cells most likely does not indicate cell death but could be a sign of postischemic cellular damage (Ünal-Çevik et al., 2004). This would be in line with the strong effect of transient OGD on CA3 network oscillations compared with CA1, hinting toward a reduced size of the recruited ensembles. The unchanged number of detected PV+ cells indicates that a full depletion of intracellular PV in its capacity as a calcium buffer did not take place and is not the main mechanism behind PV-IN dysfunction in our experiment, in line with the “interneuron energy hypothesis” (Kann, 2016).

Furthermore, we stained for the transcription factor c-Fos in PV+ and NeuN+/PV– cells (which are comprised almost exclusively of PYR in the PYR layer). c-Fos serves as an activity marker and is known to be upregulated early after HI (Kiessling et al., 1993). The c-Fos staining revealed regional differences between PV+ and PYR neurons after OGD. While OGD led to an increase of c-Fos+/PV+ cells in CA1, in CA3 c-Fos+ PYR increased. The observed levels of c-Fos expression are comparable to measurements after brief transient HI *in vivo* (Kiessling et al., 1993).

In summary, our study supports the hypothesis that FSI are functionally more vulnerable to HI insults as compared with PYR. However, in our experimental paradigm, the I/E balance after reperfusion was not significantly perturbed and the main features of SPW-R oscillations and cell-to-LFP coupling were maintained. Nonetheless, the distinct, region-specific alterations in SPW-R activity, which arose after recovery, implicate impaired information processing in the hippocampus. Impaired generation of SPW-R as observed in our study implicates scarcer information replay and thus might hamper memory consolidation. Treatments aiming to protect or restore the inhibitory function of FSI might improve network function after cerebral HI and prevent the development of lasting functional deficits.

## Synthesis

Reviewing Editor: Susanne Wolf, Charite University Hospital Decisions are customarily a result of the Reviewing Editor and the peer reviewers coming together and discussing their recommendations until a consensus is reached. When revisions are invited, a fact-based synthesis statement explaining their decision and outlining what is needed to prepare a revision will be listed below. The following reviewer(s) agreed to reveal their identity: Rainald Schmidt-Kastner. Note: If this manuscript was transferred from JNeurosci and a decision was made to accept the manuscript without peer review, a brief statement to this effect will instead be what is listed below.

The reviews about the paper were very positive and the manuscript only needs minor changes. Please find the comments below.

Reviewer 1

This paper deals with an important area surrounding recovery from hypoxia/ischemia using in vitro techniques including tetrode and whole cell voltage clamp recordings.

I think that it is important to present the details and variability of the times required to induce HSD, the variability of the HSD events, and if there is any relationship between the duration of HSD events and ensuring electrophysiological measures.

page 5: “Only slices spontaneously expressing SPW-R with amplitude > 0.2

21 mV and incidence > 1 Hz were chosen for experiments.” How many slices in all were tested and what percentage of slices fit their criteria?

Why was mannitol used once HI was evident? Mannitol could be a treatment for hypoxic-induced cell swelling.

Page 9: whole cell voltage clamp recordings. I agree with reviewer 1, point 2, that the intrinsic properties such as resting membrane potential, input resistance and threshold should be reported if available since these properties could greatly influence neuronal activity which will influence the network properties. It is also of interest if these properties were significantly different than controls yet with little effect on the network properties.

For EPSC measures, why were the holding potentials not at the stated reversal potentials for EPSPs and IPSPs?

Reviewer 2

This is certainly a very interesting study using neurophysiological methods to investigate the functional recovery of CA1 pyramidal neurons versus PV-interneurons, along with network effects, after oxygen-glucose deprivation (OGD) in adult mouse hippocampal slices. As is customary, OGD in vitro is considered as a relevant model for ischemia-hypoxia in vivo.

This experiment differs from a typical ischemia experiment in vivo where a standard vascular occlusion time is used and CA1 cells are damaged. The study used a functional parameter, the recorded occurrence of SD, to define the initial impact which variably occurred after about 6 min following the implementation of OGD. This impact was probably milder than severe ischemia for 5-10 min in vivo that damages CA1 neurons. Slices were then returned to oxygen glucose delivery and recovered after 40-60 min based on functional criteria. It is unclear from the present report for how long the recovered slices could be followed out. It cannot be excluded that some of the changes may disappear at a later time point (i.e. recovery) which cannot be studied due to the overall deterioration of the slice preparation.

The positive aspects and results of this technically sophisticated study are that PV-interneurons are functionally more vulnerable to transient OGD and SD than pyramidal cells. The authors report a shift in the I/E balance with stronger excitation (which could be relevant for the traditional concept of excitotoxicity) but then reject the hypothesized shift in I/E balance at the network level using the spike coupling to SPW-r. One would wish that in the discussion the importance of the finding is also made clear to those readers interested in hypoxia and ischemia, that are not familiar with the neurophysiological methods and terminology (which nowadays may be the majority).

A critical point is that the timing of the fixation at the end of the recordings is not provided, and one has to speculate that is was less than three hours after the OGD insult. At that time, it will be impossible in this immersion fixed slice material to make any reliable determination about some minor cell death, particularly as CA1 neurons undergo delayed neuronal death (as discussed). NeuN may not show cell death at this point. On the other hand, the stability of the PV immunofluorescence signal is important, as it may be used to argue that functional changes observed are not due to the reduction of PV related calcium-buffering. Some rewriting of the related paragraph is recommended.

Additional comments: Since SD was short and not repetitive, the concerns are not shared by this reviewer.

At some point, it should be also mentioned that PV interneurons are not the only ones providing inhibition to the pyramidal neurons, there are other classes. Is this an issue with the interpretation?

Legend to Fig. 1, under B, “representative” (replacing exemplary).

## Author Response

Dear Dr. Wolf,

thank you for the rapid and positive response to our submission. The authors thank the reviewers and the reviewing editor for their valuable comments on the manuscript. Please find below a point-by-point response to the reviews.

Reviewer 1

"This paper deals with an important area surrounding recovery from hypoxia/ischemia using in vitro techniques including tetrode and whole cell voltage clamp recordings.

I think that it is important to present the details and variability of the times required to induce HSD, the variability of the HSD events, and if there is any relationship between the duration of HSD events and ensuring electrophysiological measures.”

We thank the reviewer for her/his valuable comments. We agree with the reviewer that HSD properties and their correlation with electrophysiological outcomes are of great importance. To address this, we describe the latency to HSD and its variance in the results section (p. 14, line 300). Further, we have performed the respective additional analyses as asked for by the reviewer and added Figure 1-2 to the manuscript, which gives an overview of HSD amplitude, duration, and latency to HSD (Fig. 1-2A - C). Since our LFP tetrode recordings were high-pass filtered at 0.3 Hz by the amplifier and we judged it insufficient to evaluate SD morphology, we used data from additional DC LFP electrodes, which we used in 5 of the 25 recorded slices, for this. To test for potential effects of latency to HSD, we performed a linear regression analysis between latency and electrophysiological outcomes, i.e. the change in discharge frequency of FSI, PYR, CA1 SPW-R, and CA3 SPW-R, respectively (Fig. 1-2D - G; p. 15, lines 317 - 318; p. 18, lines 408 - 409). We did not perform a linear regression analysis of the HSD duration and outcome measures (which had been requested by the reviewer), as this data were unfortunately not available in most slices, as explained above. We hope that the additional data can provide the reader with a sufficient understanding of HSD parameters in this study. In any case, the fact that latency to HSD was not related to any of the tested outcome measures supports the assumption that using HSD as an endpoint of OGD exposure does not induce a bias in our results.

"page 5: “Only slices spontaneously expressing SPW-R with amplitude > 0.2

mV and incidence > 1 Hz were chosen for experiments.” How many slices in all were tested and what percentage of slices fit their criteria?"

Per animal, 6 slices were tested, and between 1 and 4 (usually 2) chosen for experiments. We have updated the Methods section (p. 5, lines 93 - 95, 101 - 102) to provide the reader with more detail on the slice preparation and the median number of slices used per animal. We did not systematically assess the number of slices not fulfilling the criteria, but the percentage of “good slices” (usable according to criteria) was certainly above 80%, if not 90%. We assume that in the few slices which had to be discarded neurons or long-range connections have been damaged during the preparation. Therefore, excluding of such slices is needed to avoid contamination of results with an experimental artifact.

"Why was mannitol used once HI was evident? Mannitol could be a treatment for hypoxic-induced cell swelling.”

We aimed to model the ischemia-aspect of HI, which includes insufficient glucose supply. Mannitol was used as a metabolically inactive, equimolar substitute for glucose during OGD to exclude effects of changes in osmolarity on the outcome of the study. This protocol has been previously used in several studies of OGD by different authors. The reviewer points out that mannitol could possess protective effects in HI, which is an interesting question. Several studies investigated the effects of mannitol in various in vivo and in vitro models. Indeed, it has been discussed as a hydroxyl radical scavenger (Bolanõs and Medina 1996; Almeida et al. 1998), albeit in significantly higher concentrations than in our study (50 vs. 10 mM). The same authors, however, found no protective effects of mannitol on mitochondrial oxidative stress (Almeide et al., 2002). Pre-treatment with mannitol also did not show protective effects in several hypoxia-ischemia models in vivo (Luvisotto et al., 1996) and is not being used clinically as a treatment for brain oedema in stroke. However, hyperosmolar solutions of mannitol are being therapeutically used in cases of elevated intracerebral pressure in traumatic brain injury to osmotically draw liquid from the brain. In the present study, these osmotic effects can be excluded due to equimolarity of the solutions.

"Page 9: whole cell voltage clamp recordings. I agree with reviewer 1, point 2, that the intrinsic properties such as resting membrane potential, input resistance and threshold should be reported if available since these properties could greatly influence neuronal activity which will influence the network properties. It is also of interest if these properties were significantly different than controls yet with little effect on the network properties.”

We agree that intrinsic membrane properties have an influence on network activity, as they determine a neuron’s ability to spike. The data for Figure 4 was obtained from whole cell voltage clamp recordings using a Caesium(Cs)-based intracellular solution. This allows for better space clamp properties and more accurate PSC recordings, especially of IPSC. As Cs-based intracellular solution depolarizes neurons and changes action potential properties, no current clamp recording was performed. Thus, data on resting membrane potential and action potential threshold is unfortunately not available to us. We have, however, added the data for input resistance to our manuscript (p. 17, lines 372 - 374), which is of course critical to a neuron’s excitability. Input resistance did not differ significantly between groups.

"For EPSC measures, why were the holding potentials not at the stated reversal potentials for EPSPs and IPSPs?"

We list the nominal holding potentials used for sEPSC (-75 mV) and sIPSC (+15 mV) recording. After subtraction of the calculated liquid junction potential (15 mV) they match the stated GABA (-90) and AMPA (0) reversal potentials. We have added a sentence to clarify this in the manuscript (p. 10, lines 208 - 210).

Reviewer 2

"This is certainly a very interesting study using neurophysiological methods to investigate the functional recovery of CA1 pyramidal neurons versus PV-interneurons, along with network effects, after oxygen-glucose deprivation (OGD) in adult mouse hippocampal slices. As is customary, OGD in vitro is considered as a relevant model for ischemia-hypoxia in vivo.”

This experiment differs from a typical ischemia experiment in vivo where a standard vascular occlusion time is used and CA1 cells are damaged. The study used a functional parameter, the recorded occurrence of SD, to define the initial impact which variably occurred after about 6 min following the implementation of OGD. This impact was probably milder than severe ischemia for 5-10 min in vivo that damages CA1 neurons. Slices were then returned to oxygen glucose delivery and recovered after 40-60 min based on functional criteria. It is unclear from the present report for how long the recovered slices could be followed out. It cannot be excluded that some of the changes may disappear at a later time point (i.e. recovery) which cannot be studied due to the overall deterioration of the slice preparation.

"The positive aspects and results of this technically sophisticated study are that PV-interneurons are functionally more vulnerable to transient OGD and SD than pyramidal cells. The authors report a shift in the I/E balance with stronger excitation (which could be relevant for the traditional concept of excitotoxicity) but then reject the hypothesized shift in I/E balance at the network level using the spike coupling to SPW-r. One would wish that in the discussion the importance of the finding is also made clear to those readers interested in hypoxia and ischemia, that are not familiar with the neurophysiological methods and terminology (which nowadays may be the majority).”

We thank the reviewer for his/her encouraging comments. We also agree that the mentioned paragraph might be too technical for some readers and have added a synopsis placing the electrophysiological findings in the context of HI. (p. 22, lines 502 - 503; p. 23, lines 504 - 506).

"A critical point is that the timing of the fixation at the end of the recordings is not provided, and one has to speculate that is was less than three hours after the OGD insult. “

We fixated the slices directly after recording, ∼ 1 h after the insult, which we now clarify in the methods section (p. 11, line 234).

"At that time, it will be impossible in this immersion fixed slice material to make any reliable determination about some minor cell death, particularly as CA1 neurons undergo delayed neuronal death (as discussed). NeuN may not show cell death at this point. On the other hand, the stability of the PV immunofluorescence signal is important, as it may be used to argue that functional changes observed are not due to the reduction of PV related calcium-buffering. Some rewriting of the related paragraph is recommended.”

We have edited the discussion to include the stability of the PV-signal, and its implications (p. 25, lines 573 - 574; p. 26, lines 575 - 576)

"Additional comments: Since SD was short and not repetitive, the concerns are not shared by this reviewer.

At some point, it should be also mentioned that PV interneurons are not the only ones providing inhibition to the pyramidal neurons, there are other classes. Is this an issue with the interpretation?"

We agree that the presence of non-PV-IN inhibiting PYR is certainly an important aspect and now include this in our discussion (p. 23, lines 524 - 528). We do not think that this is an issue with the interpretation, as in the context of SPW-R, PV-IN are thought to be the most relevant class of interneurons.

"Legend to Fig. 1, under B, “representative” (replacing exemplary).”

We thank the reviewer for the comment and have replaced “exemplary” with “representative”.
