# Supplementary material for: Transient Oxygen-Glucose Deprivation Causes Region- and Cell Type-Dependent Functional Deficits in the Mouse Hippocampus In Vitro
Source: eNeuro. 2021 Sep 28;8(5):ENEURO.0221-21.2021. doi: 10.1523/ENEURO.0221-21.2021 (PMC8482850; doi:10.1523/ENEURO.0221-21.2021)
Supplement: Extended Data Figure 1-1 — Parameters used in the Wave_clus algorithm. Download Figure 1-1, DOCX file. [file enu-eN-NWR-0221-21-s01.docx]

| **SPC** |  | **Interpolation** |  |
| --- | --- | --- | --- |
| par.mintemp | 0 | par.int_factor | 5 |
| par.maxtemp | 0.251 | par.interpolation | "y" |
| par.tempstep | 0.01 |  |  |
| par.SWCycles | 100 |  |  |
| par.KnearNeighb | 11 | **Features** |  |
| par.min_clus | 40 | par.min_inputs | 10 |
| par.max_clus | 200 | par.max_inputs | 0.75 |
| par.randomseed | 0 | par.scales | 4 |
| par.temp_plot | "log" | par.features | "wav" |
| par.c_ov | 0.7 |  |  |
| par.elbow_min | 0.4 |  |  |
|  |  | **Force membership** |  |
| **Detection** |  | par.template_sdnum | 3 |
| par.tmax | "all" | par.template_k | 10 |
| par.w_pre | 20 | par.template_k_min | 10 |
| par.w_post | 44 | par.template_type | "center" |
| par.alignment_window | 10 | par.force_feature | "spk" |
| par.stdmin | 5 | par.force_auto | TRUE |
| par.stdmax | 500 |  |  |
| par.detect_fmin | 300 | **Template matching** |  |
| par.detect_fmax | 3000 | par.match | "y" |
| par.detect_order | 4 | par.max_spk | 40000 |
| par.sort_fmin | 300 | par.permut | "y" |
| par.sort_fmax | 3000 |  |  |
| par.sort_order | 2 |  |  |
| par.ref_ms | 1 |  |  |
| par.detection | "neg" |  |  |
